# Revisiting coronary artery–cameral vessels: clarifying ‘MICROFISTULAE’ and moving beyond eponymous terms

**DOI:** 10.1093/omcr/omaf044

**Published:** 2025-05-28

**Authors:** Brett Thomas Snodgrass

**Affiliations:** Applied Tech, North, 1700 Derhake Rd, Florissant, MO 63033, United States

**Keywords:** coronary artery–cameral vessels, ontology, ventriculocoronary arterial connection, coronary microfistula, eponyms, coronary–cameral vessel

To The Editor

I read with great interest the article by Farandzha et al., titled ‘Coronary artery microfistulae, Thebesian veins, or vessels of Wearn?,’ published in *Oxford Medical Case Reports* [1]. Their discussion highlights a persistent conundrum in cardiovascular anatomy: the varied terminology applied to small vascular channels connecting coronary arteries to cardiac chambers. Terms such as ‘microfistulae,’ ‘arterioluminal vessels,’ ‘arteriosinusoidal vessels,’ ‘vessels of Wearn,’ and ‘Thebesian veins’ have all been employed, at times interchangeably, leading to confusion regarding whether these structures are pathologic or merely normal variants.

This commentary aims to clarify why the eponym ‘vessels of Wearn’ is not recommended for standard usage today. Instead, I advocate consistently using ‘coronary artery–cameral vessels,’ a descriptor that more accurately conveys these channels’ anatomic and embryologic nature, and propose that they be formally recognized within future international classification of diseases (ICD) classifications as either physiologically normal or ‘abnormally prominent’ when implicated in pathology.

## Background

### Microfistulae: Etymology and Evolving Meanings

‘Fistula’ originates from Latin, meaning ‘pipe’ or ‘tube’ [2]. Historically, medical sources in the 18th and 19th centuries labeled any tubular passage a ‘fistula,’ but by the early 20th century, works such as *Dorland’s* Dictionary [3] and *Gray’s Anatomy* [4] consistently defined a fistula as an abnormal or pathological passage. Applying ‘microfistulae’ to normal embryologic structures inadvertently suggests pathology [5].

### Wearn’s 1933 Observations and Later Eponyms

Wearn et al. (1933) described two subtypes of coronary artery–cameral channels: arterioluminal and arteriosinusoidal vessels [6]. Although later authors coined the term ‘vessels of Wearn’ to honor his contributions [7, 8], modern medical nomenclature increasingly discourages eponyms that fail to indicate function or typical/atypical status.

#### Histological Rationale for ‘Vessels’

Wearn carefully documented how each subtype gradually loses ‘classic’ arterial layers (media, adventitia, internal elastic lamina) as the lumen approaches the endocardium:

### Arterio-Sinusoidal Vessels

- Begin as small branches of a coronary artery.
- Progressively lose arterial characteristics, thinning to a simple endothelial tube with minimal subendothelial connective tissue.
- Walls are ‘very thin’ and the lumina are irregular, often ranging 50–250 μm in diameter.
- Because they resemble the ‘sinusoids’ described by Minot in 1900, Wearn called them ‘myocardial sinusoids’ when they open into the ventricle.

### Arterio-Luminal Vessels

- Typically retain a thicker arterial wall closer to the chamber.
- Often take a short, direct course from the coronary artery to the heart cavity, though some branch further into capillaries.
- Measured diameters (in a collapsed state) can range from 0.04 mm to 0.2 mm or up to 1.0 mm in cast preparations.
- Found communicating with both atria and ventricles, though more numerous in the ventricles.

Wearn selected the neutral term ‘vessels’ because neither subtype remains a fully characteristic ‘artery’ when examined closer to the cardiac chamber, yet nor are they veins [6]. By avoiding the term ‘artery,’ he underscored their transitional histology—a rationale that still supports using function-based rather than eponym-based nomenclature.

### Venous vs. Arterial Confusion: Thebesius, Pratt, and Vieussens

Historically, Thebesian veins refer to small venous channels emptying into the cardiac chambers, studied by Thebesius (1708) and Pratt (1898) [9, 10]. Pratt’s injection studies clarified these venous connections, but literature sometimes conflated venous ‘Thebesian’ vessels with arterial channels. Meanwhile, Vieussens (1706) was among the first to describe arterial connections within the heart [11], though these have also been confused with venous structures in subsequent texts. These arterial connections are most accurately grouped under the broader descriptor ‘coronary artery–cameral vessels.’

### Recommended Terminology: Standard Terms for Vascular Connections in the Heart

Eponyms such as ‘vessels of Wearn’ or ‘vessels of Thebesius,’ while historically significant, often fail to convey the function or nature of the structures they describe, potentially causing confusion in modern medical contexts. Instead, I propose the following standardized, descriptive terms for vascular connections between the coronary system and cardiac chambers:

- Coronary Artery–Cameral Vessels: Normal channels connecting coronary arteries directly to the heart chambers.
- Coronary Vein–Cameral Vessels: Normal channels linking coronary veins to the heart chambers.

This terminology provides multiple benefits:

1. Avoids Pathologic Implications: Unlike ‘microfistulae,’ which suggests abnormality, these terms reflect the normal anatomical presence of these connections.
2. Moves Beyond Eponyms: It aligns with the shift in medical nomenclature toward descriptive terms, reducing reliance on historical names that may not be universally recognized.
3. Supports Classification: It distinguishes these structures as normal variants that may become clinically significant in certain conditions.

Additionally, I suggest renaming ‘thebesian foramina’ as endocardial foramina. This term combines a clear noun (‘foramina’) with a precise modifier (‘endocardial’), applying to openings for both arterial and venous connections and eliminating eponymous ambiguity.

The table below summarizes the proposed terms, their definitions, and the synonyms they replace:

**Table:** 

Term	Definition	Synonyms Replaced
Coronary Artery–Cameral Vessels	Normal channels connecting coronary arteries directly to heart chambers.	Vessels of Wearn, ventriculocoronary arterial connections, arterioluminal vessels, arteriosinusoidal vessels
Coronary Vein–Cameral Vessels	Normal channels linking coronary veins to heart chambers.	Thebesian veins
Endocardial Foramina	Openings in the endocardium serving as connection points for both vessel types.	Thebesian foramina

This framework establishes a consistent, function-based nomenclature for these anatomical structures, enhancing communication and understanding in clinical and academic settings.

### Clinical Context: When Coronary Artery–Cameral Vessels Become Prominent

In conditions such as myocardial noncompaction or pulmonary atresia with intact ventricular septum (PAIVS), coronary artery–cameral vessels may enlarge and become clinically significant [12–14]. For example, in PAIVS, these vessels can form critical components of right ventricular-dependent coronary circulation [14]. Similarly, they may be prominent in cases like that reported by Farandzha et al. [1]. Recognizing these structures as normal embryologic channels that gain hemodynamic importance in specific disease states is essential for accurate diagnosis and management. Misclassifying them as inherently pathological could lead to unnecessary interventions or patient anxiety, underscoring the need for precise terminology.

6. ICD Classification Proposals.

Current ICD-10 and ICD-11 classifications often categorize coronary artery–cameral connections as congenital or acquired ‘fistulas,’ which can mislead clinicians when these channels are normal variants made visible by altered hemodynamics [15, 16]. To address this, I propose the following coding structure:

- Coronary Artery–Cameral Vessels—Not Otherwise Specified (NOS): For incidental findings without hemodynamic compromise.
- Coronary Artery–Cameral Vessels—Other Specified Condition (OSC): For cases where dilation or increased flow results in clinically significant effects, such as shunting or ischemia.

This two-tiered approach acknowledges the embryologic normalcy of these vessels while providing a distinct code for clinically relevant cases. Prominent coronary artery–cameral vessels associated with ischemic symptoms would be coded under the OSC category. In situations where the relationship between the vessels and pathology is less likely or not suspected, the code would be coronary artery–cameral vessels NOS. A similar NOS/OSC structure could be applied to ‘coronary vein–cameral vessels’ to prevent misclassification between arterial and venous connections. This coding could evolve with future research, but would lay the groundwork for disambiguation between the arterial and venous connections to the heart chambers.

## Conclusion

The term ‘microfistulae’ implies abnormality where none may exist, and eponyms like ‘vessels of Wearn’ or ‘Thebesian vessels’ risk confusing arterial and venous structures. Adopting ‘coronary artery–cameral vessels’ as a precise, descriptive term enhances clarity and aligns with the modern preference for structure-based nomenclature with functional subclassifications coded as NOS or OSC.

Future ICD revisions could incorporate codes like ‘NOS’ for normal variants and ‘OSC’ for clinically significant cases. This granularity would improve clinical decision-making, reduce misclassification, and clarify when intervention is warranted, accommodating refinements as our understanding of these vessels’ roles in pathophysiology advances.
